# An Alternative Method for Long-Term Culture of Chicken Embryonic Stem Cell *In Vitro*

**DOI:** 10.1155/2018/2157451

**Published:** 2018-05-09

**Authors:** Li Zhang, Yenan Wu, Xiang Li, Shao Wei, Yiming Xing, Zhengxing Lian, Hongbing Han

**Affiliations:** ^1^Beijing Key Laboratory for Animal Genetic Improvement, College of Animal Science and Technology, China Agricultural University, Beijing 100193, China; ^2^State Key Laboratory of Agrobiotechnology, China Agricultural University, Beijing 100193, China; ^3^National Engineering Laboratory For Animal Breeding, College of Animal Science and Technology, China Agricultural University, Beijing 100193, China

## Abstract

Chicken embryonic stem cells (cESCs) obtained from stage X embryos provide a novel model for the study of avian embryonic development. A new way to maintain cESCs for a long period *in vitro* still remains unexplored. We found that the cESCs showed stem cell-like properties *in vitro* for a long term with the support of DF-1 feeder and basic culture medium supplemented with human basic fibroblast growth factor (hbFGF), mouse stem cell factor (mSCF), and human leukemia inhibitory factor (hLIF). During the long culture period, the cESCs showed typical ES cell morphology and expressed primitive stem cell markers with a relatively stable proliferation rate and high telomerase activity. These cells also exhibited the capability to differentiate into cardiac myocytes, smooth muscle cells, neural cells, osteoblast, and adipocyte *in vitro*. Chimera chickens were produced by cESCs cultured for 25 passages with this new culture system. The experiments showed that DF-1 was the optimal feeder and hbFGF was an important factor for maintaining the pluripotency of cESCs *in vitro*.

## 1. Introduction

Since embryonic stem cells (ESCs) were first obtained from mouse embryos [1], tremendous efforts have been made to study ESCs varying from species, like human [2], zebrafish, porcine [3], and bovine [4]. The avian embryo is an excellent model for the study of embryonic development and genetic manipulation because of its accessibility. Being a nonmammalian species, the pluripotency of a chicken blastoderm at stage X was confirmed by chimera production and differentiation into various lineages [5, 6]. Pain and his colleagues maintained the cESCs (chicken embryonic stem cells) *in vitro* for a long time leading to the establishment of the cell line 9N2-5 [7, 8]. Thereafter, the cell culture methods for cESCs were improved successively by using culture medium supplemented with simplified culture recipe containing IGF1, mSCF, hIL-6, and hIL6-sR and an irradiated feeder of STO (S, SIM; T, 6-thioguanine resistant; O, ouabain resistant) cells. Buffalo rat liver-conditioned medium (BRL-CM) and STO feeders can be also used [9]. Recently, Boast and Stern described a method for culturing pluripotent blastodermal cells and differentiating them into mesoderm (bone), endoderm, and neuroectoderm (neurons and glia) in a monolayer culture [10].

Presently, appropriate cytokines and feeder layers are widely applied to keep cESCs undifferentiated. The leukemia inhibitor factor (LIF), a member of the interleukin- (IL-) 6 family, was already known to be effective in maintaining the undifferentiated state of embryonic stem cells [11, 12]. Besides, previous studies have also demonstrated that application of exogenous basic fibroblast growth factor (bFGF) could prevent hES (human embryonic stem) cell differentiation [13] and sustain undifferentiated proliferation in hES cells [14]. And bFGF played a significant role in the proliferation of chicken primordial germ cells [15]. In addition, stem cell factor (SCF) has been reported to maintain embryonic germ cells pluripotent [16]. And though the STO feeder layer with BRL-CM could maintain cESCs for over 20 passages, the cells are heterologous, the preparation of the conditioned medium is tedious, and the growth factors present in the conditioned medium are less known [9]. The STO feeder layer that was heterologous of chick combined with BRL-CM could maintain cESCs for over 20 passages. Moreover, homologous feeder layers, primary cultures of chick embryo fibroblast (CEF), and media conditioned by a chicken hepatocarcinoma line (LMH) were able to prevent the stem cell differentiation of a stage X embryo [17]. Similar to CEF, DF-1 is a continuous cell line of chicken embryo fibroblasts [18]. As described above, DF-1 has the potential to be an optimal feeder layer for maintaining cESCs.

In order to establish a stable and simple culture system of avian embryonic stem cells, we clarified the appropriate conditions for the cESC culture *in vitro*, including the growth factors and the feeder layers. Under the optimal culture conditions, we have followed the protocol used to identify the cESC characteristics originally described by Pain et al. to verify this culture system [8].

## 2. Materials and Methods

### 2.1. cESC Culture

The basic culture medium was high glucose DMEM (Gibco, UK) supplemented with 2 mM glutamine, 1 mM sodium pyruvate (Gibco, UK), 0.16 mM *β*-mercaptoethanol (Sigma, USA), 100 U/ml penicillin (Gibco, UK), and 100 *μ*g/ml streptomycin (Gibco, UK). The complete medium refers to the basic culture medium complemented with growth factors 20 ng/ml hLIF (R&D Systems, USA), 20 ng/ml mSCF (R&D Systems, USA), 20 ng/ml hbFGF (R&D Systems, USA), and 5% fetal bovine serum (FBS) (Gibco, UK).

The pCEF cells were derived from 10-day-old White Leghorn embryos. The STO cells were purchased from China Center for Type Culture Collection, and the DF-1 cells were purchased from the Type Culture Collection of the Chinese Academy of Sciences, Shanghai, China. The cells were cultured at 37°C in 5% CO_2_ in the basic culture medium supplemented with 10% fetal bovine serum. The pCEF cells and the DF-1 cells were treated with 20 *μ*g/ml of mitomycin C (Sigma) for 60 minutes at 37°C in 5% CO_2_. And the STO cells were treated with 20 *μ*g/ml of mitomycin C (Sigma) for 120 minutes at 37°C in 5% CO_2_. Feeders were prepared at a density of 10^5^ cells/cm^2^.

The blastodermal cells were isolated from freshly laid eggs of White Leghorn chicken. The quality of embryos at stage IX-XI is vital to the success of the whole experiment. The entire blastoderm was harvested as mentioned previously and then dissociated in tubes containing PBS at room temperature [19]. After the cells were washed twice with PBS and then centrifuged at 1000 rpm, 5 min, they were resuspended in the complete culture medium using gentle aspiration. Finally, they were seeded onto the feeder cells in 96-well plates at a final concentration of 1.5 × 10^4^ cells per well. Each day, half of the complete culture medium was replaced. After 5–7 days, when the blastoderm cells appeared to be the typical rounded, clone-like, phase-bright morphology of ES cells, the feeder cells nearly died; the blastoderm cells were allowed to passage into 1.5–2 wells. This procedure was repeated until the requisite amount of cells to perform experiments was obtained. It is recommended that the cells be treated with Ca^2+^/Mg^2+^-free PBS for 2-3 minutes prior to passaging, as this will allow the cESCs to be easily passaged and resuspended into single cells while the differentiated cells are left behind.

### 2.2. Alkaline Phosphatase Reaction

The cultured cells were fixed with cold 4% paraformaldehyde at 4°C for 30 minutes. Then the cells were gently washed thrice with PBS. The freshly prepared alkaline phosphatase staining solution (100 mM NaCl, 100 mM Tris–HCl pH 9.5, 5 mM MgCl_2_, 1 mg/ml NBT, and 0.1 mg/ml BCIP) was added to the fixed cells. The cells containing the solution were then incubated for 5–30 minutes at room temperature, and then, after removing the solution, it was washed with PBS. Colored colonies were visible when the culture was viewed using an inverted microscope (Nikon, Japan).

### 2.3. High-Content Analysis

Cultured cells were fixed with cold 4% paraformaldehyde for 30 minutes at 4°C. The paraformaldehyde was then aspirated from the plates, and the cells were washed thrice with PBS. The cells were then blocked with a blocking buffer (10% goat serum in PBS) at room temperature for 1 hour. The primary antibodies SSEA-1 (Abcam, 1 : 100) and Nanog (Abcam, 1 : 150), both of which were diluted in the blocking buffer, were incubated along with the fixed cells overnight at 4°C. After washing the cells thrice, the cells were then incubated with the secondary antibodies CY5 (Abcam, 1 : 500) and Texas Red (ZSGB-Bio, 1 : 200), which were also diluted in the blocking buffer, for 1 hour at room temperature. The EdU proliferation detection was performed as per the described protocol (C10812-3, Ribobio). The cells were then washed 3 times and observed, and a high-content analysis was performed (ImageXpress Micro XLS Widefield High-Content Analysis System, USA). The proliferation rate is equal to the positive EdU-Nanog coexpression cell number over the positive Nanog expression cell number.

### 2.4. RT-PCR and Quantitative Real-Time PCR

Total RNA was extracted from different passage cells by using RNeasy Mini Kits (Qiagen, Germany), and then cDNA was synthesized using Thermo Scientific RevertAid First Strand cDNA Synthesis Kit (Thermo, USA). Gene expression levels were quantified using GoTaq® qPCR Master Mix (Promega, USA) and Mx3000P qPCR System (Agilent Technologies, USA). The relative gene expression levels were quantified using the formula: △△Ct = (Ct of the target gene − Ct of *β*-actin) treatment − (Ct of the target gene − Ct of *β*-actin) control. *β*-Actin is the housekeeping gene. All primer pairs used are listed in Table S1.

### 2.5. Transmission Electron Microscopy (TEM)

Specimens were cut into approximately 1 mm cubes and fixed in 2%-2.5% glutaraldehyde for 2 hours at 4°C. Then the samples were washed with PBS (pH 7.2) and fixed in 1% osmium tetroxide. Subsequently, the specimens were washed in sodium cacodylate buffer and dehydrated with gradient alcohol. After that, the specimens were immersed in propylene oxide and embedded in Epon 812. Then the cubes were cut into semithin sections (1 *μ*m) and stained with methylene blue for localization under a microscope. Ultrathin sections obtained by cutting were stained with uranyl acetate and lead citrate. Finally, the samples were examined under a JEM-1400 electron microscope.

### 2.6. Telomerase Activity

Cells were harvested at passages 0, 3, 5, and 20. HCT8 cells were collected for the positive control. Subsequently, the cell pellets (1 × 10^6^ cells) were washed once with ice-cold Ca^2+^/Mg2^+^-free PBS and once with ice-cold telomeric repeat amplification protocol (TRAP) washing buffer. Then, a lysis buffer with a volume that is 4-5 times the volume of a cell pellet was added. The cell pellets were incubated on ice for 30 minutes with occasional agitation, followed by centrifugation at 18,000*g* for 30 minutes at 4°C. Then the PCR-based TRAP assay, as described in the publishment by Huawei Xin, was performed. The primer pairs used are shown in Table S. 15 *μ*l out of 50 *μ*l PCR products was electrophoresed for 1 hour at 100 V using 12% polyacrylamide gel. The PCR-amplified fragments were developed by rapid silver stain for nucleic acid (Tiandz, China).

### 2.7. Embryoid Body (EB) Formation

The growth factors bFGF, SCF, and LIF were removed from the complete culture medium approximately 6 days after the cESCs were passaged without dissociating cells. At that point, almost all the feeder cells died and ES-like colonies were formed. One half of the medium was changed every 2 days in the following 1-2 weeks until the EB morphology occurred.

### 2.8. In Vitro Differentiation of Cultured cESCs

Based on improvised methods from prior studies, we induced the directional differentiation of the cESCs into cardiac myocytes [20], osteoblasts [21], nerve cells [22], smooth muscle cells [23], and adipocytes. All of the cESCs used to perform directional differentiation were in the 20th passage and the induction was initiated 6 days after subculturing, and at that time, almost all the feeder cells died and the cESCs aggregated well. The protocols for differentiation of chick avian ES cells into mesodermal and neuroectodermal derivatives are summarized in the supplemental experimental procedures. The antibodies used to identify various cell types are shown in the supplemental experimental procedures (Supporting Information S3) as well.

### 2.9. Oil Red O Staining

For fat cell detection, Oil Red O staining was performed. After the cells were fixed by 4% PFA, the fixative solution was removed and the cells were washed 3 times with PBS. The staining solution was prepared fresh as mentioned: 6 parts of Oil Red O stock solution, 0.5 g Oil Red O (Sigma, USA) in 100 ml isopropanol, and 4 parts of distilled water. Next, the staining solution was filtered using the Whitman paper and was then incubated with the culture cells for 20 min. Finally, the staining solution was removed and the cells were thoroughly washed with PBS 3 times.

### 2.10. Alizarin Red Staining

For osteoblast detection, Alizarin Red staining was performed. The 0.1% staining solution was prepared as stated: 0.1 g Alizarin Red S (Amresco, USA) in 100 ml distilled water, with a pH value adjustment of 4.1~4.3. The culture cells were stained with the Alizarin Red solution for 20 to 30 minutes, and the reaction was observed microscopically. Then the staining solution was removed and the cells were washed with PBS 3 times to get the clear picture.

### 2.11. In Vivo Differentiation of Cultured cESCs

The injection into the recipient embryo was performed as described by Perry [23] and Cao et al. [24]. Newly laid fertilized Shouguang chicken eggs, obtained from the ranchette of China Agricultural University (Beijing, China), were used as a recipient for the cells which were derived from White Leghorn chicken eggs and then cultured for over 25 passages *in vitro* with the support of our culture system (more information in the supplemental experimental procedures). A 24 bp insertion/deletion mutation (5′-ACAAGAAGAGACAAGACAAGGAAG-3′) exists in the PRLpro2 gene. Different chicken breeds exhibit distinct genotype frequency distribution of PRLpro2. In order to identify whether the donor cells have contributed to the development of chicken embryos in the recipient, a PCR reaction was performed. Day 8, 10, 12, 13, and 15 embryos were used for the extraction of DNA by the traditional phenol/chloroform procedure. PCR amplification conditions are as follows: PRLpro2 primer (Table S) (94°C for 5 min), followed by 30 cycles of amplification (94°C for 30s, 57°C for 30 s, and 72°C for 30 s), followed by 72°C extension for 5 min. PCR products were analyzed using 3% agarose gel.

### 2.12. Statistical Analyses

The statistical significance of the differences observed in samples was determined using the Wilcoxon rank sum test or the Student two-tailed *t*-test. Data were shown as the means ± SD of at least 3 independent experiments. *P* < 0.05 was considered to be statistically significant.

## 3. Results

### 3.1. Optimal cESC Culture Condition

**Figure 1:**
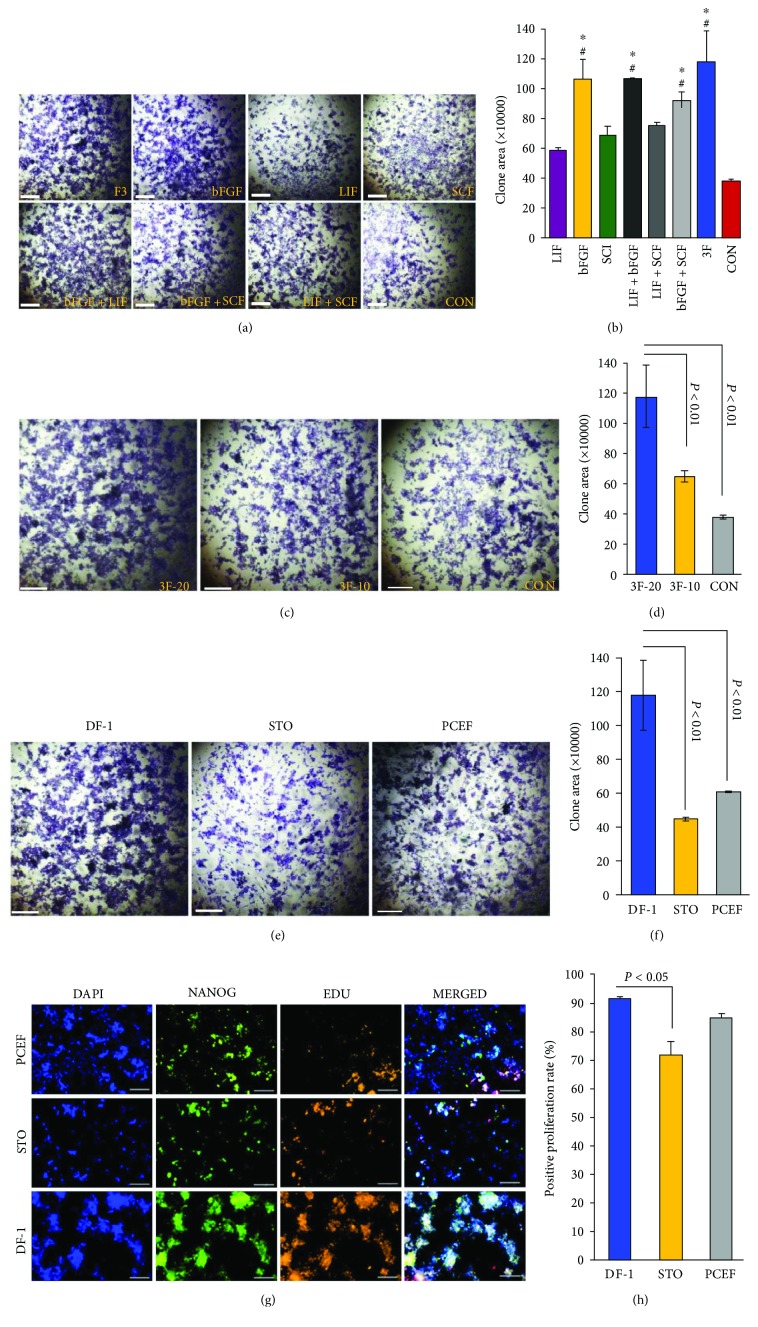
The effect of feeders and factors on the development of avian blastodermal cells. (a) bFGF is vital to the culture of avian blastodermal cells *in vitro*. cESCs were plated in the presence of or in the absence of growth factors as indicated in the figure. Crystal violet staining was performed after 4 passages. (b) The areas of colonies with crystal violet staining were counted by Image-Pro Plus. ^∗^Compared to the control group, there is a significant difference (*P* < 0.05). ^#^Compared to the LIF, SCF, and LIF + SCF groups, there is a significant difference (*P* < 0.05). (c) The combination of 20 ng/*μ*l each of hLIF, hbFGF, and mSCF was the optimal concentration. cESCs were plated in 0, 10, and 20 ng/*μ*l each of hLIF, hbFGF, and mSCF, respectively. Four passages later, crystal violet staining was performed. (d) The areas of colonies were calculated by Image-Pro Plus. The colony area in the presence of 20 ng/*μ*l growth factors was the biggest (*P* < 0.01). (e) The inactivated DF-1 feeder was the optimal feeder for supporting cESCs *in vitro* compared to STO and pCEF feeders. (f) The areas of colonies with crystal violet staining after 4 passages were counted by Image-Pro Plus. The colony area on the DF-1 feeder was the biggest (*P* < 0.01). (g) The cultured cESCs after 4 passages on different feeders were performed by DAPI (blue), EDU (green), and NANOG (orange) staining. Scale bar: 150 *μ*m. (h) The bars indicated the positive proliferation rate of the cultured cESCs after 4 passages on different feeders. Cells on the DF-1 feeder and pCEF feeder showed the highest proliferation rate (*P* < 0.05). All results are expressed as the mean ± SD of three independent experiments. Scale bar: 100 *μ*m.

We followed the cell culture protocol described by Pain and his colleagues [17]. The chicken embryonic stem cells obtained from stage X embryos were plated at a final concentration of 1.5 × 10^4^ cells per well in a basic culture medium with FBS on DF-1 feeder layers. Different growth factors were then added to the basic culture medium. Four passages (around 25 days) later, cells were fixed and stained with crystal violet (Figure 1(a)). The colonies showing a compact and round morphology were observed. We analyzed the clone area by Image-Pro Plus.  Figure 1(b) displays the necessity of bFGF for the cESC culture *in vitro*. The area of colonies in culture with the addition of additives (hLIF, mSCF, and hbFGF) significantly increased compared to that with the addition of hLIF, mSCF, or hbFGF separately (*P* < 0.05). In comparison to lower concentrations of cytokine (10 ng/ml), a combination of 20 ng/ml each of hLIF, hbFGF, and mSCF showed a greater impact on the area of colonies (Figures 1(c) and 1(d)). When comparing the clone growth on different types of feeders, the inactivated DF-1 feeder provided the most regular ES-like morphology, the largest colony area (*P* < 0.01) (Figures 1(e) and 1(f)), and the highest ratio of Nanog-positive proliferation cells (*P* < 0.05) (Figures 1(g) and 1(h)). Thus, these data demonstrated that the DF-1 feeder was the optimal feeder cell for the culture of cESCs *in vitro*. Meanwhile, basic culture medium complemented with 20 ng/ml hLIF, hbFGF, and mSCF was identified as the complete culture medium for the following experiments.

**Figure 2:**
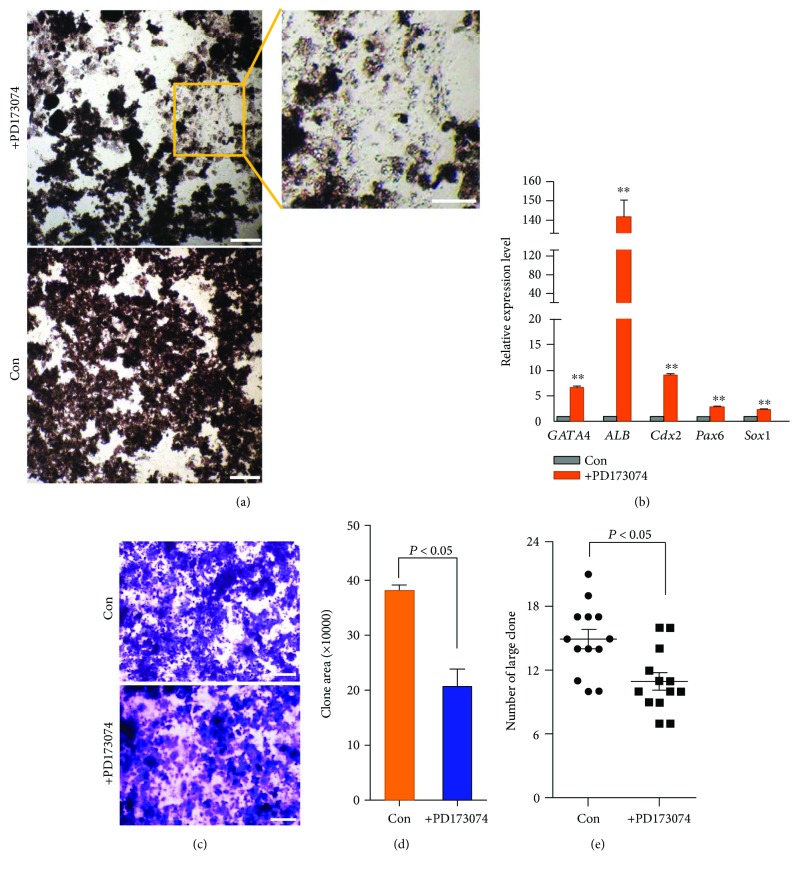
bFGF inhibits the differentiation of cESCs *in vitro*. (a) The inhibition of FGFR1 promoted the differentiation. The primary cESCs in the complete medium were treated with an effective FGFR1 inhibitor PD173074. Differentiated cells were highlighted in the yellow box. (b) The inhibition of FGFR1 led to an increase in the mRNA level of three germ layer marker genes. ^∗∗^implies: compared to control group, there is a extremely remarkable difference (*P* < 0.01). Quantitative RT-PCR of GATA4, ALB, CDX2, PAX6, SOX1, and *β*-actin was performed. (c) The inhibition of FGFR1 suppressed the formation of the clone. Crystal violet staining of primary cESCs in the presence with PD173074. (d) The colony area stained by crystal violet was calculated by Image-Pro Plus (*P* < 0.05). (e) The Image-Pro Plus analysis of the clone number (*P* < 0.05). All results are expressed as the mean ± SD of three independent experiments. Scale bar: 100 *μ*m.

The results revealed that the cESCs expressed FGFRs (fibroblast growth factor receptors) (Figure S2) and the hbFGF significantly increased the area of colonies, compared to hLIF and mSCF (Figure 1(b)). To further identify the effect of hbFGF, PD173074 (an effective FGFR1 inhibitor) was added in the complete medium to effectively inhibit fibroblast growth factor receptor 1 (FGFR1). The cESCs were differentiated partially by FGFR inhibition (Figure 2(a)). To further check whether bFGF has the function to inhibit differentiation, transcripts of marker genes related to all three germ layers were examined by quantitative real-time PCR. The result suggested that cESCs were prone to differentiate when bFGFR was blocked (Figure 2(b)). Crystal violet staining revealed that when the cESCs were treated with the PD173074, the clone was smaller (*P* < 0.05) (Figures 2(c) and 2(d)) and the clone number was less (*P* < 0.05) (Figure 2(e)). These data suggested that bFGF could inhibit differentiation of cESCs and thus facilitated the maintenance of the cESCs *in vitro*.

### 3.2. Identification of cESCs In Vitro

**Figure 3:**
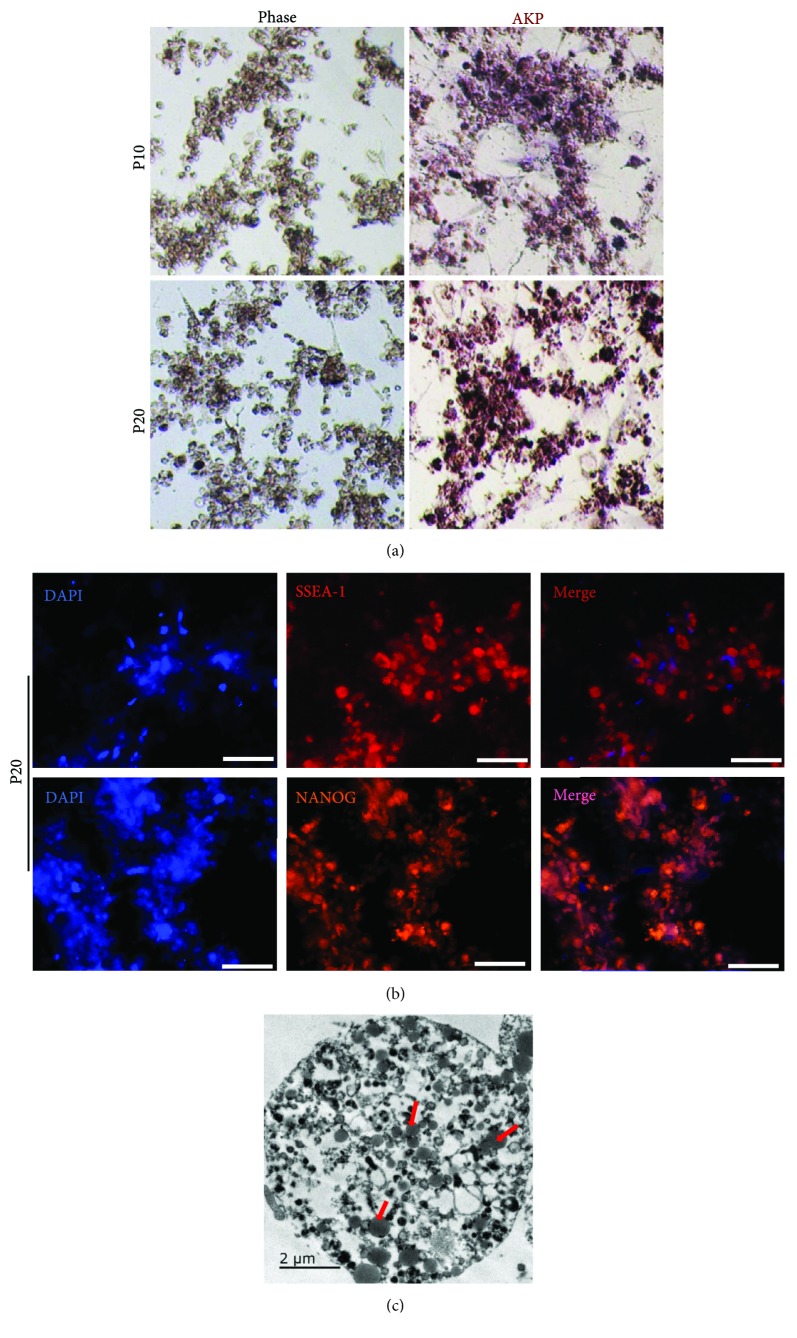
Identification of cESCs *in vitro*. (a) cESCs in the complete culture system showed alkaline phosphatase activity over 20 passages. (b) Pluripotency marker profile of cultured cESCs after 20 passages. Scale bar: 100 *μ*m. (c) The transmission electron microscopy (TEM) photos of cESCs and red arrows indicated the lipid droplets. Scale bar: 2 *μ*m.

The positive reaction with endogenous alkaline phosphatase has been shown previously to be the first criterion to confirm nondifferentiated embryonic stem cells [8]. The cESCs which had 20 passages still showed the positive staining of alkaline phosphatase and expression of stem cell markers SSEA-1, Nanog (Figures 3(a) and 3(b)). Furthermore, we found electron-lucent lipid-rich droplets (red arrow in Figure 3(c)) with a round or oval shape and different sizes less than 2 *μ*m in the cESCs, and then the results were identical with the findings described by Li and colleagues [25]. The transmission electron microscopy (TEM) photos indicated that the cytoplasm of cESCs is filled with lipid droplets. Moreover, cESCs with pluripotency potential were still observed after 48 passages (over 260 days; figure not shown).

### 3.3. Characteristics of cESCs in Long Culture Period

**Figure 4:**
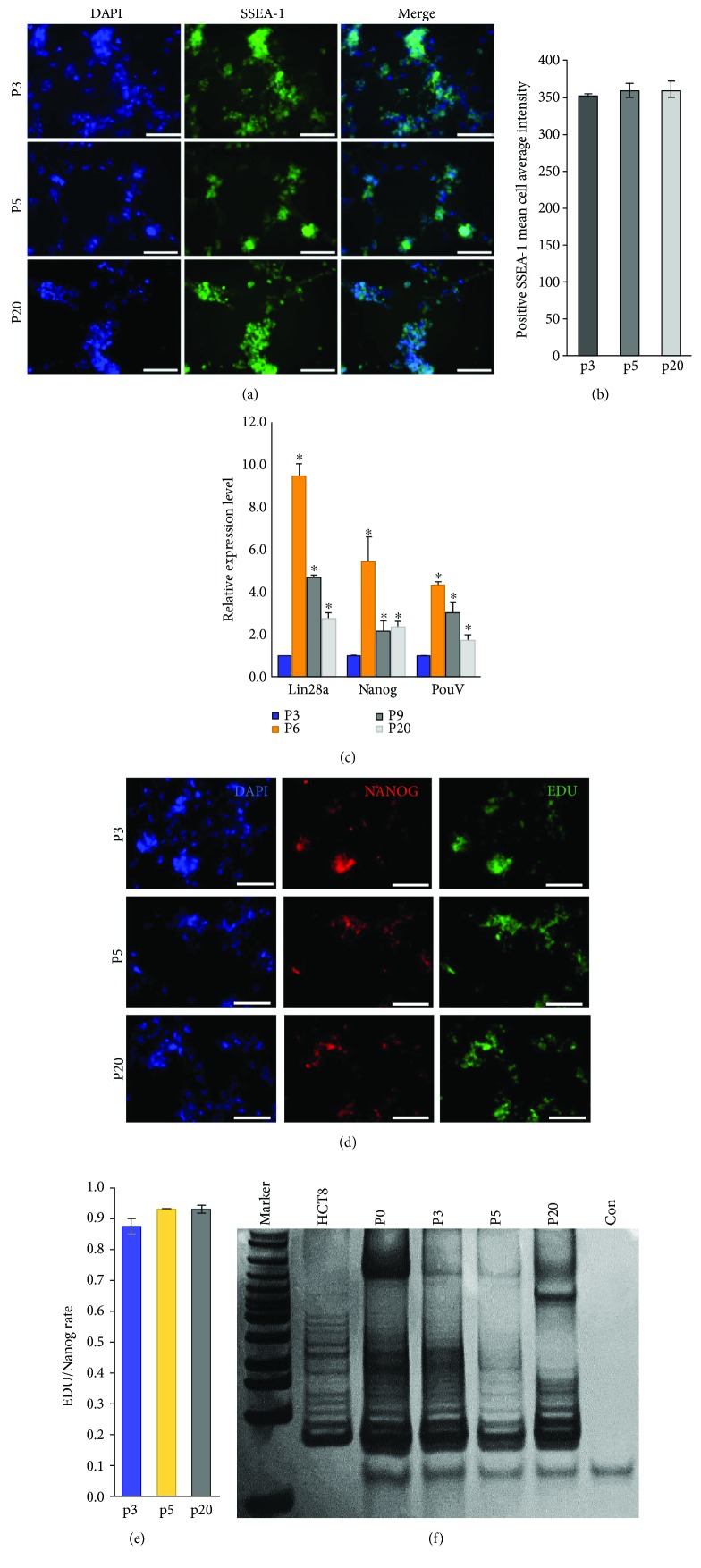
Characteristics of cESCs in a long culture period. This culture system can stably maintain the stem-like character of cESCs *in vitro* for a long term. (a) Epitope characteristic of cultured cESCs in passages 3, 5, and 20. The pluripotency surface marker SSEA-1 (green) was stained. Meanwhile, DAPI (blue) was used to stain the nucleus. (b) SSEA-1 expression was maintained at a relatively stable level. The ImageXpress Micro XLS Widefield High-Content Analysis System was used. The bars indicated the positive SSEA-1 mean cell average intensity of cultured cESCs in passages 3, 5, and 20. (c) *Lin28a*, *Nanog*, and *PouV* gene mRNA relative expression level of cultured cESCs in passages 3, 6, 9, and 20. ^∗^Compared to the expression level of cultured cESCs in passage 3, *P* < 0.05. (d) Nanog expression characteristic of cultured cESCs in passages 3, 5, and 20. The figure indicated the merged picture of DAPI, Nanog, and EDU staining. (e) The positive proliferation rate of cultured cESCs was maintained at a relatively high level over passages 3, 5, and 20. The bars indicated the ratio of the EDU-Nanog coexpression cell number to the Nanog expression cell number calculated by ImageXpress Micro XLS Widefield High-Content Analysis System. (f) Telomerase activity. Telomerase activities of cultured cESCs in passages 0, 3, 5, and 20 were measured in cell lysate by the TRAP assay. The cells were frozen until the TRAP was performed. HCT8 cell lysate used as a positive control and noncell lysate used as a negative control. DNA ladder (N3233 New England Biolabs) was used to indicate the size of the fragment. All results are expressed as the mean ± SD of three independent experiments. Scale bar: 100 *μ*m.

To verify the properties of cESCs maintained in our new system, markers of pluripotency and proliferation were monitored. In passages 3, 5, and 20, the positive proliferation rate and SSEA-1 expression were detected. The SSEA-1 expression level was relatively stable along with the long-term cell culture (Figures 4(a) and 4(b)). At the same time, we detected the expression levels of *Lin28a*, *Nanog*, and *PouV* of cESCs in passages 3, 6, 9, and 20 (Figure 4(c)). These pluripotency marker genes were maintained at a relatively high expression level. Immunofluorescence of EDU and Nanog showed that the proliferation rate remained at around 90% (Figures 4(d) and 4(e)). Telomerase activity was an important marker of stem cells because of its high level in ES cells and cancer cells. cESCs were collected at different passages to test telomerase activity using TRAP (telomerase repeat amplification protocol) assay. The result indicated that the telomerase activity of passages 0, 3, 5, and 20 displayed 6 bp periodicity (Figure 4(f)). The high level of telomerase activity expressed by cESCs maintained in this culture system was similar to that of undifferentiated stem cells. The relatively constant activity in different passages indicated this new culture condition could prevent cESC differentiation during long-term cultures *in vitro*. These cells were characterized with expressions of stem cell markers, the typical morphology of ES cell-like colony, and high telomerase activity. Given these observations above, this new culture system can maintain the stem-like character of stage X avian blastoderm cells for a long term *in vitro*.

### 3.4. In Vitro Differentiation of Cultured cESCs

**Figure 5:**
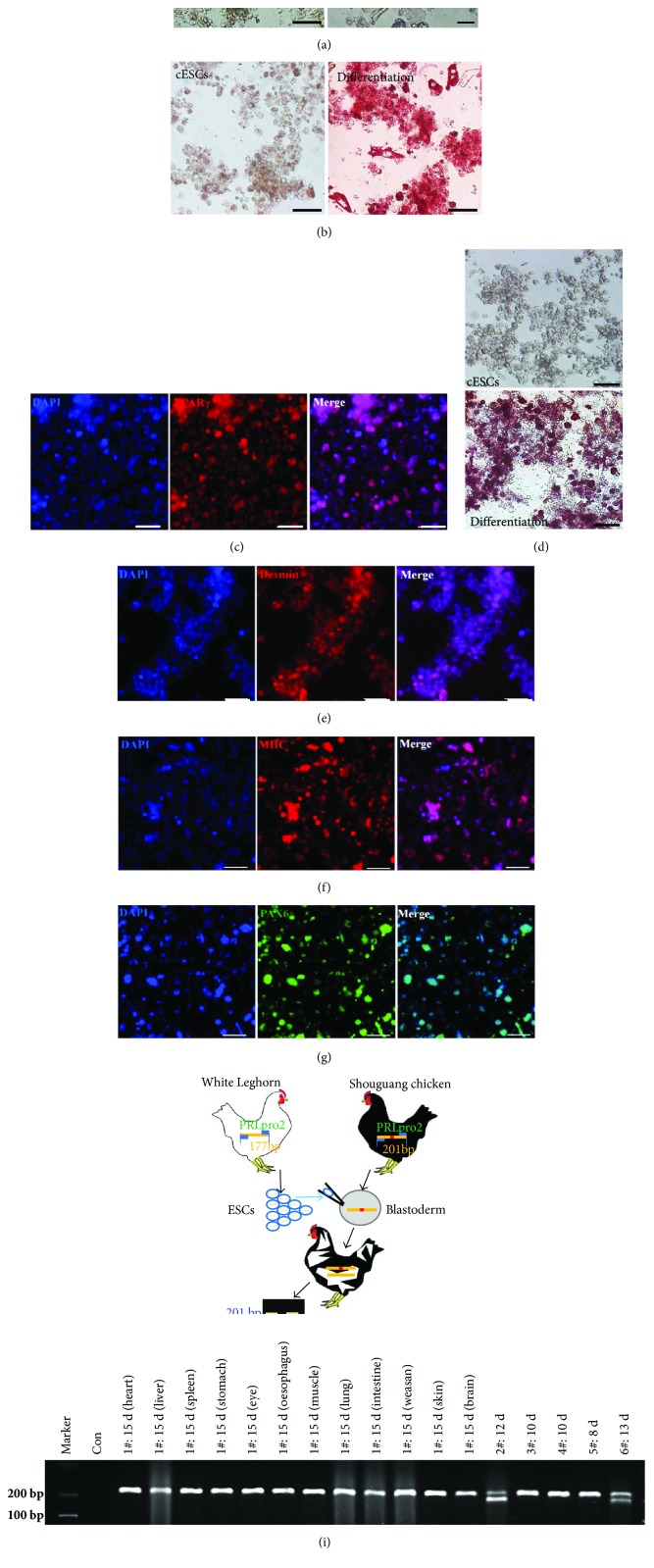
*In vitro* and in vivo differentiation of cultured cESCs. (a) Embryoid body-like structures in the phase after the cESCs were cultured without the growth factors for 10 days. (b) Oil Red O positive staining showed the differentiation to adipocytes in the phase. cESCs with complete culture medium in the phase were the control. (c) The expression of PPAR*γ* (red) confirmed the differentiation to adipocytes, and the nucleus was stained by DAPI (blue). (d) Alizarin Red staining verified the potentiality to osteoblasts. cESCs with complete culture medium in the phase were the control. (e) The immunofluroscence of desmin (red) and DAPI (blue) certified the directional differentiation to smooth muscle cells. (f) MHC (red) and DAPI (blue) staining proved differentiation to cardiac myocytes. (g) The immunofluorescence of PAX6 (green) and DAPI (blue) showed the differentiation to neural cells. (h) The process of chimeric chicken production was indicated in the figure. Chimeric chickens generated after grafting cultured cESCs of 25 passages *in vitro*. (i) The two distinct genotypes of *PRLpro2* on the gel picture verified that there were two successful productions of chimeric embryos. PCR analysis of *PRLpro2* genotype. Six chicken embryos after 15 d, 12 d, 10 d, 8 d, and 13 d incubation were tested. Scale bar: 100 *μ*m.

The ability to differentiate into various lineages is a hallmark of stem cells. We developed embryonic bodies form several passages of cultured cESCs (see Materials and Methods). The floating organised structures like embryonic bodies occurred after being cultured without bFGF, SCF, and LIF about 10 days (Figure 5(a)). To test whether cESCs could be induced to differentiate into multiple lineages i*n vitro*, the directional differentiation as described in the experimental procedures was performed on cESCs of different passages. Four weeks later, by Oil Red O staining and immunofluorescence of adipocyte marker PPAR*γ* [26], we verified that cESCs could differentiate into adipocyte as judged with typical morphology containing many intracellular lipid droplets (Figures 5(b) and 5(c)). As shown in Figure 5(d), some cells along with osteoblasts markers were revealed by staining with Alizarin Red four weeks after induction of the cultured cells. We were able to identify the expression of Desmin and MHC, the markers of cardiac myocytes and smooth muscle cells, respectively. The results revealed that cESCs could differentiate into both cell types originating from the mesoderm (Figures 5(e) and 5(f)). Besides, the employment of neural cell-specific marker PAX6 [27] showed that the cESCs could generate mature neurons (Figure 5(g)). These results suggested that the stem-like cESCs were able to differentiate into mesoderm and neuroectoderm lineages and that the method described above is efficient for maintaining the pluripotency of cESCs *in vitro*.

### 3.5. In Vivo Differentiation of Cultured cESCs

In order to further verify the pluripotency potential of the cultured blastodermal cells, we injected the cESCs cultured for 25 passages into recipient subgerminal cavity of stage X Shouguang Chicken embryos to produce the chimeras (Figure 5(h)). The cultured cESCs were harvested from White Leghorn and maintained on the DF-1 feeder in a completed culture medium with 5% FBS. Since many embryos died in the early incubation period, we only checked 6 embryos incubated for 8, 10, 12, 13, and 15 days. Different chicken breeds exhibit distinct genotype frequency distribution of *PRLpro2* [28]. An isolated organ of an embryo incubated for 15day was tested to identify the *PRLpro2* genotypes. Unfortunately, there was no chimeric expression pattern. Meanwhile, we tested the organ mixture of 5 embryos to see the *PRLpro2* genotypes, and two embryos incubated for 12 and 13 days showed the chimeric expression of the *PRLpro2* gene (Figure 5(i)). In this way, we certified the successful production of the chimeras and further verified that the cESCs can be maintained by this new culture system *in vitro* for a long time with pluripotency.

### 3.6. Discussion

The aim of this work was to establish a stable and simple culture method to maintain avian blastodermal cells with ES features *in vitro*. Avian pluripotent stem cells have the ability to generate both the germ line and the somatic chimeras [29]. The progress in the culture of avian pluripotent stem cells has been remarkable in recent years. So far, only the production of germ line chimeras of avian ES from early passages is feasible [30, 31]. Accordingly, an optimal culture condition for maintaining the pluripotency of cESCs for a long term is in demand.

Our results suggested that this newly established culture system is able to maintain the blastodermal cells with stem-like features over 48 passages (more than 260 days) *in vitro*. The round and “ES-like” shape of the cell clusters was confirmed as the cells, which have the pluripotency potential by the endogenous alkaline phosphatase staining and the immunofluorescence of SSEA-1. The cell cluster is round in shape but not as round and compact as human or mouse embryonic stem cells. They do not have the defined boundaries that the mES and hES cells have. Chicken embryonic stem cells are nonadherent and round in shape, with a round nucleus and a low cytoplasm-to-nucleus ratio. Also, cESCs showed the ability to differentiate into cardiac myocytes, smooth muscle cells, neural cells, osteoblasts, and adipocytes. While some cultured cESCs may have the ES morphology and epitope profile, the most stringent assay for testing a developmental potential is to verify the ability to generate somatic and germ line chimeras. Inspiringly, blastodermal cells cultured from 25 passages in our newly established system were able to yield chimeras, whereas Pain's study reported that blastodermal cells cultured from 1–3 passages were capable of generating somatic chimeras. Our study showed that the chimeras were produced by injecting the late passage cESCs. Given that the culture of cESCs should make the selection of cells which have integrated the transgene possible and would allow cells to undergo targeted recombination events, defining the optimal conditions to maintain pluripotency for a relatively long term is crucial for generating transgenic chickens by using these cESCs. Beyond the generation of transgenic chickens, the cESC technology could be used to shorten the time needed for conventional poultry breeding programs and for *in vitro* human therapeutic protein production [24].

Our culture system is simple since it consists of the homologous feeder layer, DF-1, and only 3 cytokines, hbFGF, mSCF, and hLIF. According to the results, it is still capable of maintaining the pluripotency of cESCs for a long term. Even though the mRNA expression level of the pluripotency marker in passage 20 was downregulated compared to early passages 6 and 9 (Figure 4(c)), the expression level was similar to that of stage passage 3. The mRNA expression of pluripotency varied within an accepted range. Beyond this, telomerase activity was an important marker of stem cells because of its high level in ES cells and cancer cells. The relatively constant telomerase activity in different passages indicates that this new culture condition could prevent cESC differentiation in long-term cultures *in vitro*. Besides, chimeras were produced by cESCs cultured for 25 passages with this new culture system. The stable cell growth allows us to investigate the important factors involved in the pluripotency maintenance leading to lay a solid theoretical foundation for the application of cESC technology.

The culture of stem cells on the feeder cell layer is regarded as a good strategy of supporting stem cells since the feeder cell layer secretes an extracellular matrix containing some factors into the culture medium. These factors help with the attachment and survival, promote self-renewal, and suppress the differentiation of stem cells [32]. In our study, both heterologous and homologous feeder layers were tested. Dissociated cells from the fresh chicken blastoderm at stage X were initially cultured with STO feeder layers, primary chick embryonic fibroblast (CEF) feeder layers, or the DF-1 feeder layer which is an immortalized cell line of chicken embryo fibroblasts transformed from a 10-day chicken embryo. A notable result is that the morphology of cESC clones is better and the clone area is bigger on the DF-1 feeder layer compared to STO and pCEF. Meanwhile, the positive proliferation rate of cESCs is higher on pCEF and DF-1 than on STO. It is our assumption that because DF-1 and pCEF cells are both avian homologies, the cytokines released are more suitable for cESCs than cytokines from the STO cells. Moreover, it is easier to produce inactivated feeder cells using an immortalized cell line and the effect of DF-1 is much more stable than the primary cells [33]. For a long time, the DF-1feeder layer greatly facilitates studies on oncogenic transformation and cell killing by avian viruses [34]. It is the first time to be identified as an optimal feeder layer for culturing cESCs.

However, none of the above feeder cells alone could maintain the blastodermal cells beyond two passages. When the combination of feeder cells and 3 cytokines was used to culture blastodermal cells, the pluripotency of cells could be maintained. The three cytokines, hLIF, hbFGF, and mSCF, are known to retain the pluripotency and proliferation potential of human and mouse stem cells [35, 36]. However, their roles in avian stem cells have not yet been fully clarified. Our results indicate that among these three cytokines, bFGF had a significant effect on the cESCs. The cESCs have a better growth state and a longer undifferentiated state with the culture medium complemented with bFGF. Therefore, the importance of bFGF was further investigated in our study. In this study, we detected the expression of FGFRs in cESCs, which indicated that the bFGF might make an effect on cESC maintenance. When PD173074 was added in the culture system, the cESCs compact and round began to be adherent and transparent, distinctly. Our results revealed that the inhibition of FGFRs pushed cESCs toward differentiation. As for human stem cells, the inhibition of the FGF receptor also increased differentiation but had a little effect on the cell number. Moreover, FGF receptor functionality in human ES cells is indirect [37]. In addition, it is proved that FGF-2 is associated with cell proliferation as well. For instance, exogenous FGF-2 stimulated the expression of genes in human stem cell and also suppressed cell death and apoptosis. There exists a niche in the hESC that FGF-2 interacts cooperatively with IGF-2 to maintain self-renewal [38]. And bFGF was stabilized by cell surface heparan sulfate which could further promote human ES cell proliferation [39]. It is found that bFGF activates the MEK/ERK cell signaling pathway and stimulates the proliferation of chicken primordial germ cells [15]. While we observed that bFGF inhibits differentiation, the mechanism of bFGF in maintaining pluripotency of cESCs in our culture system needs further clarification.

In our experiment, stage X avian blastoderm cells with stem cell morphology and epitope profiles could be maintained with stem-like characteristics for a long term *in vitro* with the support of the DF-1 feeder and basic culture medium supplemented with only three cytokines: human basic fibroblast growth factor (hbFGF), mouse stem cell factor (mSCF), and human leukemia inhibitory factor (hLIF). The alternative simple method *in vitro* was used to maintain the pluripotent capability of avian blastodermal cells. These encouraging findings provided a starting point for further refinement and eventual progress in the application of cESC technology.

## Data Availability

The data used to support the findings of this study are available from the corresponding author upon request.

## Supplementary Materials

Supplementary Materials Table S: the primers for PCR, Q-PCR, and TRAP in experiments. Figure S2: expression of bFGF, FGFR1, FGFR2, FGFR3, and FGFR4 for feeder cells and cESCs, respectively, using RT-PCR. Supporting Information S3: supplemental experimental procedures including in vitro differentiation of cultured cESCs, in vivo differentiation of cultured cESCs, and crystal violet staining.
