# Supplementary material for: An Alternative Method for Long-Term Culture of Chicken Embryonic Stem Cell In Vitro
Source: Stem Cells Int. 2018 May 9;2018:2157451. doi: 10.1155/2018/2157451 (PMC5971340; doi:10.1155/2018/2157451)
Supplement: Supplementary Materials — Table S: the primers for PCR, Q-PCR, and TRAP in experiments. Figure S2: expression of bFGF, FGFR1, FGFR2, FGFR3, and FGFR4 for feeder cells and cESCs, respectively, using RT-PCR. Supporting Information S3: supplemental experimental procedures including in vitro differentiation of cultured cESCs, in vivo differentiation of cultured cESCs, and crystal violet staining. [file 2157451.f1.doc]

**Supplemental Information**

Table S. The primers for PCR, Q-PCR and TRAP in experiments.

Figure S2. Expression of bFGF, FGFR1, FGFR2, FGFR3, FGFR4 for feeder cells and cESCs, respectively, using RT-PCR.

S3. Supplemental experimental procedures including in vitro Differentiation of cultured cESCs, in vivo differentiation of cultured cESCs and crystal vioviolet staining.

Table S. The primers for PCR, Q-PCR and TRAP in experiments.

| **Name** | **Forward primers** | **Reverse primers** | **Type** |
| --- | --- | --- | --- |
| LIN28A | CACCTTCAAGAAATCCTCCA | GGCTCTTGCTCTTAGGTCTC | Q-PCR |
| NANOG | CTACATTTCACCAGGGCTTCC | AAAGAAGCCCTCATCCTCCA | Q-PCR |
| POUⅤ | AATGAGGCAGAGAACACGGA | CACACATTTGCGGAAGAAGC | Q-PCR |
| ALB | TGCCCAGTATCTCCAGAGGT | CATTCAGGAGCATCTTCATTG | Q-PCR |
| GATA4 | TTTCTCAGGCATTCTCGGTCTC | GACTGGCTGATGGCTGACTG | Q-PCR |
| PAX6 | TAGTAAGCCGAGAGTAGCGAC | GACCCCCTCCGAGAGTAATC | Q-PCR |
| SOX1 | GCCGAGTGGAAGGTGATGTC | TGAGCAGCGTCTTGGTCTTC | Q-PCR |
| CDX2 | CAAAACCAGGACGAAGGACA | TCTGGAACCAGATTTTCACCT | Q-PCR |
| β-actin | GAGAAATTGTGCGTGACATCA | CCTGAACCTCTCATTGCCA | Q-PCR |
| TS | AATCCGTCGAGCAGAGTT |  | TRAP |
| CX | CCCTTACCCTTACCCTTACCCTAA |  | TRAP |
| PRLpro2 | GGTGGGTGAAGAGACAAGGA | TGCTGAGTATGGCTGGATGT | PCR |
| bFGF (FGF2) | TCTGGCTATGAAGGAGGATG | CCTTTTCAGTGCCACATACC | PCR |
| FGFR1 | AGTGGGATGTTTACCTGGAG | AGAAGTAGGTGGTCTCGCTC | PCR |
| FGFR2 | CAGGAGAACCACTTGAGTTG | GAATCTCTGGGTGAAGCATC | PCR |
| FGFR3 | CAAGGCATTCCAACGAGGTC | ATCTGACGGCACAACGCTCT | PCR |
| FGFR4 | CCTCTCCTACCAGTCAGCGT | CCTCTCCTACCAGTCAGCGT | PCR |

**
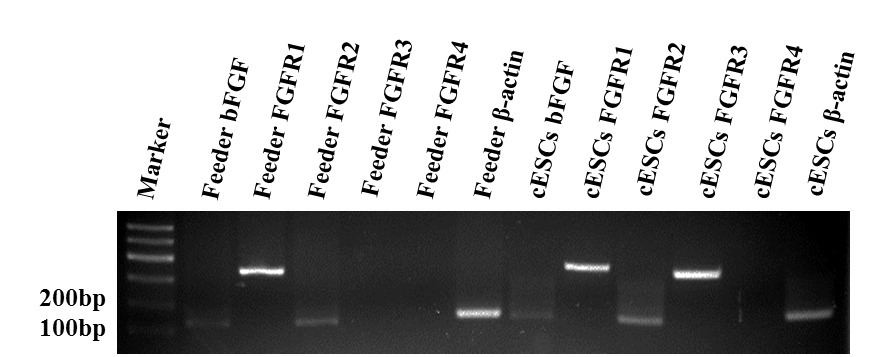
**

**Fig. S2.** Expression of FGFRs: RT-PCR for bFGF, FGFR1, FGFR2, FGFR3, FGFR4 of feeder cells and cESCs, respectively. *β-actin* is the housekeeping gene. We examined the expression of FGFRs using RT-PCR. Both cESCs and feeder cells expressed bFGF and its receptor, which indicated that the bFGF might play a role on maintaining pluripotency and self-renewal of cESCs. It is noteworthy that cESCs expressed FGFR1-3, only except for FGFR4. Nevertheless, the feeder cells merely expressed FGFR1 and FGFR2.

**S3. Supplemental Experimental Procedures**

**In vitro Differentiation of cultured** **cESCs**

Cells over 20 passages can be induced to directional differentiate into various cell types, such as adipocytes, nerve cells, smooth muscle cells, cardiac myocytes, and osteoblasts and so on. The complete cultivation medium was replaced with the differentiation medium approximate 6 days after passage. At that time, the feeder cells nearly died and cESCs aggregated. We practiced a modified version of a standard method for cardiac myocytes culture and differentiation [1]. The medium was supplemented with 0.1% dimethyl sulphoxide (DMSO, Sigma, America), 5 µM 5-azacytidine (Sigma, America), 0.1 µM Vitamin C (Sigma, America) and 10% fetal bovine serum (Gibco, UK). Half of the medium was changed every 2 days.

As for differentiating cESCs into osteoblasts, we modified a method designed for mouse ES cells [2]. When the feeder cells nearly died out, the original medium was withdrawn and replaced with essential culture medium supplemented with 0.1µM dexamethasone (sigma, America), 10 mM β-glycerophosphate (sigma, America), 50µg/ml Vitamin C and 10% fetal bovine serum (Gibco, UK). The medium changes every 2 days.

To initiate neural differentiation, the method used was developed based on protocol designed for mouse ES cells [3]. The differentiation medium was comprised essential culture medium supplemented with 5 µg / ml insulin (sigma, America), 30 nM triiodothyronine (T3, sigma, America), 20 nM hydrocortisone (sigma, America), 20 nM progesterone (sigma, America), 10 µg / ml bovine albumin (sigma, America) and 10% fetal bovine serum (Gibco, UK). Cultures were fed by partial medium replacement every 2 days.

We have followed the protocol originally described to obtain smooth muscle cells [4], with minor modifications. The primary medium was replaced by the differentiation culture medium at day 6 after passaging, which was supplemented with 10 uM retinoic acid (Sigma, America), 1 mM 1-thioglycerol (MTG, Sigma, America) and 15% fetal bovine serum (Gibco, UK). The process was sustained for 10 days with fresh media substitution every 2 days. The differentiation medium was replaced by a another medium, which was composed of essential culture medium, 1 mM MTG (Sigma, America) and 15% fetal bovine serum (Gibco, UK). The cultures were sustained for another 10 days with partial medium replacement every 2 days.

In terms of directional differentiation to adipocytes, we improves on the basis of previously study [5]. The cells were first treated with induction medium for 4 days. This medium consists of essential culture medium supplemented with 20 nM insulin (sigma, America), 1 nM triiodothyronine (T3, sigma, America), 0.125mM indomethacin (sigma, America), 5 µM dexamethasone (sigma, America), 0.5mM 3-isobutyl-1-methylxanthine (IBMX, sigma, America ) and 10% selected fetal bovine serum (Gibco, UK). Medium was changed every 2 days. Subsequently, induction medium was substituted by differentiation medium which was composed of cultivation medium supplemented with 20 nM insulin (sigma, America), 1 nM triiodothyronine (T3, sigma, America) future differentiation. Cultures were fed every 2d by partial medium replacement.

Various differentiation medium’ composition as well as the specific time of medium change were summarised as follows:

| A. Differentiation timetable for cardiac myocytes | | |
| --- | --- | --- |
| Day | 1～28 | |
|  | differentiation medium | |
|  | composition | final concentration |
|  | DMEM high |  |
|  | FBS | 10% |
|  | dimethyl sulphoxide (DMSO) | 0.10% |
|  | 5-azacytidine | 5uM |
|  | Vitamin C | 0.1uM |
|  |  |  |
| B. Differentiation timetable for osteoblasts | | |
| Day | 1～28 | |
|  | differentiation medium | |
|  | composition | final concentration |
|  | DMEM high |  |
|  | FBS | 10% |
|  | dexamethasone | 0.1μM |
|  | β-glycerophosphate | 10 mM |
|  | Vitamin C | 50 μg/ml |
|  |  |  |
| C. Differentiation timetable for neural cells | | |
| Day | 1～28 | |
|  | differentiation medium | |
|  | DMEM high |  |
|  | insulin | 5 µg/ml |
|  | triiodothyronine(T3) | 30 nM |
|  | hydrocortisone | 20 nM |
|  | Progesterone | 20 nM |
|  | bovine albumin | 10 µg/ml |

D. Differentiation timetable for smooth muscle cells

| Day | 1～10 | | 11～35 | |
| --- | --- | --- | --- | --- |
|  | differentiation medium | | culture medium |  |
|  | composition | final concentration | composition | final concentration |
|  | DMEM high |  | DMEM high |  |
|  | FBS | 15% | FBS | 15% |
|  | 1-thioglycerol(MTG) | 1mM | 1-thioglycerol (MTG) | 1mM |
|  | retinoic acid | 10μM |
| E. Differentiation timetable for adipocytes | | |  |  |
| Day | 1～4 | | 5～28 | |
|  | Induction medium | | differentiation medium | |
|  | composition | final concentration | composition | final concentration |
|  | DMEM high |  | DMEM high |  |
|  | FBS | 10% | FBS | 10% |
|  | insulin | 10% |
|  | triiodothyronine(T3) | 1nM | insulin | 20nM |
|  | indomethacin | 0.125mM |
|  | dexamethasone | 5µM | triiodothyronine(T3) | 1nM |
|  | 3-isobutyl-1-methylxanthine (IBMX) | 0.5mM |

To detect the results of directional differentiation, we with the help of the following [diverse](app:ds:diverse) antibodies:

| **Name** | **Species** | **Dilution** | **Company** | **Catalog#** |
| --- | --- | --- | --- | --- |
| PPARγ | Rabbit | 1:100 | Bioss(China) | bs-4590R |
| MHC | Chicken | 1:40 | DSHB(America) | ALD-58 |
| Desmin | Chicken | 1:40 | DSHB(America) | D76 |
| PAX6 | Chicken | 1:40 | DSHB(America) | PAX6 |

**In vivo differentiation of cultured cESCs**

The assay of injecting cultured cESCs into recipient embryo was conducted according to the work of Perry [6] and Dainan Cao [7]. Newly laid Shouguang Chicken eggs obtained from the ranchette of China Agricultural University (Beijing, China) were used as recipient for the White Leghorn cultured cells. The eggs were soaked for 3 minutes at 40°C in 0.1% of the benzalkonium bromide solution. Approximately 1–2 μl containing 100-500 cells were injected into the sub-germinal cavity of blastoderm using a microscopic manipulator (Eppendorf, Hamburg, Germany). Embryos were then incubated with a 90° rotation every 30 minutes at 60-70% relative humidity (RH) and 37.5°C in the following 3 days once transferred into surrogate shells. On day four, the well-grown embryos were placed into fresh and bigger surrogate shells derived from an egg 25g heavier than the primary. The embryos were then incubated at 37.5°C and 60–70% RH for approximately 17 days, with a 30° rotation every 30 minutes. On day 20, the embryos were transferred to a hatcher for further incubation.

**Crystal vioviolet staining**

Fix the cultured cells for 30 minutes at 4°C with 4% paraformaldehyde. Aspirate the fix solution from plates and wash the cells 3 times with PBS. Cover the cells with 0.5% crystal violet solution in 25% methanol. Incubate for 10 minutes at room temperature. Remove the crystal violet, wash the cells in PBS several times, until the dye stops coming off and the view is clear.

**References**

[1] T. Takahashi, B. Lord, P. C. Schulze, et al., "Ascorbic acid enhances differentiation of embryonic stem cells into cardiac myocytes," *Circulation*, vol. 107, no. 14, pp. 1912-1916, 2003.

[2] J. Kawaguchi, P. J. Mee and A. G. Smith, "Osteogenic and chondrogenic differentiation of embryonic stem cells in response to specific growth factors," *Bone*, vol. 36, no. 5, pp. 758-769, 2005.

[3] M. Kim, A. Habiba, J. M. Doherty, et al., "Regulation of mouse embryonic stem cell neural differentiation by retinoic acid," *Dev Biol*, vol. 328, no. 2, pp. 456-471, 2009.

[4] H. Huang, X. Zhao, L. Chen, et al., "Differentiation of human embryonic stem cells into smooth muscle cells in adherent monolayer culture," *Biochem Biophys Res Commun*, vol. 351, no. 2, pp. 321-327, 2006.

[5] M. F. Klein J, M Ito, BB Lowell, M Benito and CR Kahn., "β3-Adrenergic Stimulation Differentially Inhibits Insulin Signaling and Decreases Insulin-induced Glucose Uptake in Brown Adipocytes.," *J Biol Chem*, vol. 274, pp. 34795-34802, 1999.

[6] M. M. Perry, "A complete culture system for the chick embryo," *Nature*, vol. 331, no. 6151, pp. 70-72, 1988.

[7] D. Cao, H. Wu, Q. Li, et al., "Expression of recombinant human lysozyme in egg whites of transgenic hens," *PLoS One*, vol. 10, no. 2, pp. e0118626, 2015.
